# The Equiprobability Bias from a Mathematical and Psychological Perspective

**DOI:** 10.5709/acp-0163-9

**Published:** 2014-12-31

**Authors:** Nicolas Gauvrit, Kinga Morsanyi

**Affiliations:** 1 CHArt (Human and Artificial Cognition lab), École Pratique des Hautes Études, Paris, France; 2 School of Psychology, Queen’s University Belfast, Northern Ireland

**Keywords:** equiprobability bias, subjective probability, complexity, randomness, uniformity

## Abstract

The equiprobability bias (EB) is a tendency to believe that every process in which randomness is involved corresponds to a fair distribution, with equal probabilities for any possible outcome. The EB is known to affect both children and adults, and to increase with probability education. Because it results in probability errors resistant to pedagogical interventions, it has been described as a deep misconception about randomness: the erroneous belief that randomness implies uniformity. In the present paper, we show that the EB is actually not the result of a conceptual error about the definition of randomness. On the contrary, the mathematical theory of randomness does imply uniformity. However, the EB is still a bias, because people tend to assume uniformity even in the case of events that are not random. The pervasiveness of the EB reveals a paradox: The combination of random processes is not necessarily random. The link between the EB and this paradox is discussed, and suggestions are made regarding educational design to overcome difficulties encountered by students as a consequence of the EB.

## Background

Research on heuristics and biases (e.g., Gilovich, Griffin, & Kahneman, 2002) has documented a broad range of systematic errors in how people make decisions about uncertain events. Nevertheless, this research program has been criticized for being overly descriptive (e.g., Gigerenzer & Gaissmaier, 2011). For instance, although more than 100 scientific papers have been published on the classical conjunction fallacy (Tversky & Kahneman, 1983) over the past 30 years, the psychological bases of this striking fallacy are still debated (see e.g., Tentori & Crupi, 2012; Wedell & Moro, 2008 for recent reviews). Other theorists have pointed out that some of the “mistaken intuitions” of people about uncertain events are closer to the probabilistic characteristics of sequences of random events in real-life settings than we have ever thought (Hahn & Warren, 2009).

The purpose of the present paper is to provide an analysis of the equiprobability bias (see a definition and examples below), a persistent error in probability judgments. The equiprobability bias (henceforth EB)^1^ is a tendency to assume that every process related to randomness must be uniform (i.e., that each possible outcome has the same probability). The EB has been linked to the naďve idea that randomness implies uniformity (Lecoutre, 1992). Following on this suggestion, researchers (e.g., Anway & Bennett, 2004; Callaert, 2004; Morsanyi, Handley, & Serpell, 2013) have discussed the EB as an example of a mathematical “misconception” about randomness. Here we will argue that this is not the case. Although the EB might lead to reasoning errors, it is based on a sound mathematical assumption about randomness. The EB arises when this sound assumption is used outside its scope, with non-random processes.

Indeed, there is a perplexing property of randomness which might lead to reasoning errors: that when random processes are combined, the outcome might be in part, or, indeed, fully deterministic. In our analysis below, we will support these statements by discussing the mathematical theories of randomness and probability. Finally, we will give an overview of the existing psychological research on the EB, and highlight some important gaps in our current knowledge. Ultimately, our aim is to present some classic tasks from a new perspective, and, through this, to inspire novel psychological and educational approaches to the study of randomness and probability.

## Introducing the equiprobability bias

When two fair dice are thrown, the probability of getting a sum of 11 (one 5 and one 6) is twice as much as that of getting 12 (two 6s), because the first case may emerge from two different patterns (5-6 or 6-5), whereas the second corresponds to a unique pattern (6-6). However, people show a strong tendency to believe that 11 and 12 are equally likely. This example is a classic task in which the EB shows up. It is a variant of the historical *Galileo and the Duke of Tuscany Problem*, which dates back to the early 17th century.

The EB was first described by Lecoutre (1992) as a tendency for individuals to believe that any random variable is “equiprobable” (i.e., uniform) by nature—that is, all random outcomes share the same probability. This intuition about randomness leads to pervasive errors in solving probability problems involving non-uniform random variables, from childhood through adolescence and to adulthood (e.g., Batanero, Serrano, & Garfield, 1996; Chiesi & Primi, 2009; Falk & Lann, 2008; Lecoutre, 1992; Morsanyi et al., 2013). Strangely, the EB seems to increase with probability education, and has recently been described as a “side-effect of education” (Morsanyi, Primi, Chiesi, & Handley, 2009). Indeed, it has long been recognized that education can lead to strong intuitions (e.g., Fischbein, 1987; Fong, Krantz, & Nisbett, 1986; Stavy & Tirosh, 2000), which might be based on important insights. Nevertheless, these intuitions are sometimes misapplied by students, and can result in typical mistakes (see e.g., Morsanyi & Szucs, 2014 for a recent review).

Many authors have hypothesized about the psychological reasons for this permanent fallacy. However, to the best of our knowledge, they did not address the EB from a mathematical perspective. Consequently, there has also been no attempt to integrate the mathematical and psychological perspectives into a common framework, which could help both researchers and educators. As we will see below, there are good mathematical reasons to believe that randomness is uniform by nature. Addressing the EB from a mathematical perspective gives a fresh view on this “bias”. We will argue that the EB is not an emanation of our imperfect mind, but the result of a mathematical paradox. Although it does lead to systematic errors in probabilistic reasoning, it is based on a fundamentally correct assumption. Specifically, the EB describes the tendency for people to allocate equal probabilities to uncertain events, unless they have strong reasons^2^ to believe that some of these events are more likely than others (see also Fox & Levav, 2004; Johnson-Laird, Legrenzi, Girotto, Legrenzi, & Caverni, 1999).

## A broad variety of tasks unveil the EB

Participants exhibit the EB in the case of various tasks. In the following sections we present examples of some typical tasks, which have been found to elicit the intuition of uniformity. Note that most of these tasks were not specifically designed to elicit or demonstrate the EB, and existing studies have mostly discussed these tasks in isolation, without reference to other similar tasks (but see Falk & Lann, 2008; Morsanyi et al., 2013 for exceptions). Our aim in this section is to present a series of tasks which can be used to demonstrate a tendency in participants to erroneously assign equal probabilities to different outcomes. At the same time we also try to identify some typical characteristics of these tasks, and the factors that lead to the mistaken intuition of uniformity.

### A simple case of assigning probabilities to outcomes generated through a random process: The raffle problem

In the simplest cases, a random variable is given which is, by definition, non-uniform. An example, the raffle problem, can be found in Morsanyi et al. (2009, 2013):

A class has nine boys and six girls in it. The teacher does a raffle. Each pupil’s name is written on a slip of paper. All the slips are put in a hat. The teacher picks out one slip without looking. Who do you think is going to win the prize?

a) A boy [correct answer]

b) A girl

c) Both are equally likely [equiprobability]

As we can see in this example, a uniform random process (selecting the winner from the set of pupils through a raffle) determines a non-uniform random event (sex of the winner). Although there is a higher number of boys in the class, the reader might have the impression that this is irrelevant, given that there are only two possible outcomes, and the actual outcome is determined through a fair (i.e., random) procedure (see further discussion of these considerations by participants in, e.g., Falk & Lann, 2008; Morsanyi et al., 2009; Pratt, 2000). Indeed, Morsanyi et al. (2009, 2013) found that some university students answered “equally likely” to this task (see also the data presented in Table 1).

**Table 1.** The Rate of Equiprobability and Correct Responses from the Morsanyi et al., 2013 Study

	Random generators training (*n*=55)		Control (*n*=53)	
	Equally likely	Correct	Equally likely	Correct
Raffle problem	4%	91%	9%	89%
Two children problem	89%	5%	96%	2%
Hospital problem	51%	42%	79%	8%
Hospital problem	48%	46%	61%	33%

*Note*. The students either participated in a training with random generators or no training^5^. Percentages do not always add up to 100 because of a third response option that a few participants chose.

Fox and Rottenstreich (2003) asked participants to estimate the probability that “Sunday will be hotter than any other day next week” or that “The hottest day of the week will be Sunday”. With the first presentation, participants tend to answer “50%”, based on the partition between two possible events (Sunday is the hottest day/ Sunday is not the hottest day), whereas the second presentation prime a 1/7 answer, based on a partition into seven possible events (Monday is the hottest day, Tuesday is the hottest day, etc.). Thus, different answers may arise from the same idea that at the root of any random process lies equiprobability.

### More complex cases of assigning probabilities to randomly generated outcomes

#### Two children problem

A somewhat more complex situation is built on the confusion between ordered and unordered pairs of objects. One example is Gardner’s (1954) *two children problem*:

“Mr. Smith has two children. At least one of them is a boy. What is the probability that both children are boys?”

Every ordered pair, namely (girl, boy), (boy, girl) and (boy, boy) are equally likely, with a probability of 1/3 (consequently, 1/3 is the probability of (boy, boy) and thus also the correct answer to the two children problem). However, unordered pairs {girl, boy} and {boy, boy} are not equally likely—the probabilities being respectively 2/3 and 1/3. Because of the EB, participants often believe that unordered pairs share the same probability, and give the incorrect answer of “0.5” to the two children problem, based on the idea that {girl, boy} and {boy, boy} constitute fair alternatives. (See Table 1. for some data on the frequency of this error).

#### The three-cards problem

Falk and Lann (2008) interpreted the *three cards problem* in terms of equiprobability. This teaser is related to the notorious Monty Hall dilemma (see e.g., Baratgin & Politzer, 2010; Krauss & Wang, 2003; Petrocelli & Harris, 2011 for a psychological perspective): Three events (cards or doors) are presented, which have equal probabilities. Then a given information discards one of the three possible events. The resulting probabilities of the two remaining possibilities are not equal anymore, although participants show a tendency to assume uniformity.

In the three cards problem, 3 cards are given. One is green on each side (GG), one is red on each side (RR), and the last one is green on one side and red on the other (RG). The three cards are shuffled; a card is randomly chosen and put on a table. The participant then sees the random side of a random card, which is red. The question is: what is the probability that this card is the RR one? Typically, subjects adhere to uniformity and answer “0.5”, that is, they claim that the two possible cards (RR and RG) are equally likely. The correct answer, however, is different. Since 3 sides of the two possible cards are red, and the RR card has two red sides, the actual probability is 2/3. Once again, there is some uniformity here: The sides of the cards all share the same probability. But when combining sides to build sets of cards, we lose this feature.

#### The problem of three prisoners

Now consider the following (and mathematically equivalent) problem:

Three men, A, B and C, were in jail. C knew that one of them was to be set free and the other two were to be executed. But he did not know who was to be spared. To the jailer who did know, C said, “Since two out of the three will be executed, it is certain that either A or B will be, at least. You will give me no information about my own chances if you give me the name of one man, A or B, who is going to be executed.” Accepting this argument after some thinking, the jailer said “B will be executed.” Thereupon C felt happier because now either he or A would go free, so his chance had increased from 1/3 to 1/2. This prisoner’s happiness may or may not be reasonable. What do you think?

Similarly to the three cards problem, in the *three prisoners problem* (Lindley, 1971; Shimojo & Ichikawa, 1989) people have the impression that after discarding one event, the probability of the two remaining possibilities will be 1/2. Nevertheless, the elimination of one possibility does not provide additional information on the probability that C will be spared, which, thus, remains equal to 1/3. (At the same time, the probability that A will be spared now equals 2/3).

The problem of three prisoners as well as the three-cards problem are equivalent to the famous *Monty Hall dilemma*. This teaser involves three doors, say A, B and C. Behind one of the doors is a reward (a car) the participant will keep should s/he ultimately pick the right door. The participant first selects a door, for instance, C. Then the experimenter opens a not selected door (say B) which has no reward behind it. After this, the participant may either stick to his first choice (C) or switch to the other unopened door (A)^3^. The optimal strategy is to switch, for the same reason as in the problem of three prisoners.

A simple explanation may be given using a series of six situations with equal probability: In the first two situations, the reward lies behind door A. In the two next, the reward lies behind door B, and in the two last behind door C. If the reward is behind door A, the experimenter has no other choice than designating door B, and vice versa. If the reward is behind door C on the other hand, he can either show door A or B, which he will do once each. Among the 6 situations, the experimenter will therefore show door B three times. Among these three times, two correspond to a situation in which the reward is behind door A. Therefore, changing to door A grants a probability of 2/3 to win the reward. Not doing so yields a probability of only 1/3 to win.

However, many people think that switching or staying are equivalent, based on the false assumption that doors A and C are still equally likely to hide the reward. Baratgin and Politzer (2006, 2007, 2010) provide a theoretical explanation of the equiprobability answer in such contexts. According to their studies, contextual cues prompt different interpretations of the revision context (i.e., how the new probabilities should be computed once a new piece of information becomes available). In the context of the Monthy Hall dilemma, an “updating” procedure is prompted, which leads to an equiprobability answer (Baratgin, 2009).

### Combining random processes: variants of the two dice problem

Similarly to the two-dice problem that we described above, some tasks involve the combination of two uniform variables. For instance, the *statistical reasoning assessment* (Garfield, 1998; 2003) uses three such tasks out of the four tasks that may be used to assess the EB. In the case of the two dice problem, each die is assumed to be *fair*, another term for *uniform*. Here again, the non-uniform variable (the sum of the two dice) is determined by uniform variables (i.e., the outcome of throwing the dice).

### Size of the sample space: The hospital problem

The classic hospital problem from Tversky and Kahneman (1974) may also be used to assess the EB. Here is a version of this problem used by Morsanyi et al. (2013):

One hospital has an average of 50, another 10 births per day. The average number of boys and girls being born on each day is equal. How often will each hospital expect more than 60% boys on a given day?

a) The small hospital can expect this to happen more often than the large one [correct answer]

b) This will happen more often in the large hospital

c) It is equally likely to happen in both hospitals [equiprobability]

Here again, there is a uniform variable (sex of the babies), from which a more complex situation is built up. In this task participants might have the impression that the situation in the large hospital can be understood as a “multiplication” of the events taking place in the small hospital. Based on this argument, there would be no reason to suppose that the properties of the small and large samples should be any different.

To summarize the above sections, we have presented several typical problems which elicit the intuition of equiprobability/uniformity. Note that all of these problems include uniform variables or random processes. Crucially, in these problems, uniform processes are combined with other uniform processes, they go through some transformation, or participants fail to spell out all relevant possibilities, which then leads to the mistaken intuition of equiprobability of the potential outcomes.

## Good reasons to believe in uniformity

Noticeably, the very tasks that are aimed at proving that people make wrong assumptions about randomness when they expect random processes to be uniform, use, as a rule, some kind of underlying uniformity. This fact might give an indication of how inherent the intuition of uniformity to our conceptualisations of probability and randomness is. Moreover, this is not specific to psychological studies: Uniformity is also a constant implicit in the classroom, as well as a basic assumption in early theories of probability (see Hacking, 1975, chapter 14). Most exercises and examples in probability textbooks also use prototypical random processes or random “tools”, such as fair dice, raffles, determination of the sex of new-borns, or coin tosses. In all of these cases, we expect students to assume uniformity.

### Uniformity: an implication of randomness

Studies about naďve participants’ concepts of randomness usually show a similarity to expert views. Randomness is linked to variability, uncertainty, unpredictability, and complexity. All of these properties mentioned by ordinary participants are in line with probability theory (e.g., Lahanier-Reuter, 1999).

Another characteristic, often mentioned by participants, is uniformity (Nickerson, 2002). It may seem that uniformity is not at all a formal requirement of randomness. Indeed, probability theory defines a *random variable* as a variable corresponding to any probability distribution. In the same manner, a so-called *random event* may have any probability between 0 and 1. Nevertheless, we must here warn against a possible misunderstanding: *Random variables* and *random events* cannot be taken as definitions of randomness. Actually, even fully deterministic events (1=1, always true) or variables (*X*=1) are misleadingly called “random” in probability theory.

This issue has been noted and discussed by mathematicians for a long time. They have built theories of randomness at the boundary of probability theory and computer science (Downey & Hirschfeldt, 2010; Martin-Löf, 1966; Muchnik, Semenov, & Uspensky, 1998) to overcome this issue. We will discuss this point further.

### Equiprobability as an implicit rule in the classroom

The current theory of probability defines a probability *P* on a sample space *U* as a measure complying with the axiom of *P*(*U*) = 1. For a finite sample space *U* = {*a*_1_, …, *a*_n_}, this amounts to set non-negative numbers *P*(*a*_i_) which sum up to 1. For instance, in the situation of a coin toss with two possible outcomes, *H* and *T*, we have *U* = {*H*, *T*}. To define a probability, we can choose any number *p* between 0 and 1, and set *P*(*H*) = *p*. *P*(*T*) is then bound to equate 1-*p*, because the sum *P*(*H*) + *P*(*T*) must be 1. The usual case, in which *P*(*H*) = *P*(*T*) (i.e., *p* = 0.5) is only one of an infinity of possibilities. The other values for p may be interpreted as resulting from tossing a hypothetical *unfair* coin (note that Gelman & Nolan (2002) argued that in fact there is no such thing as an unfair coin).

However, the first definition of probability given in the classroom is Laplace’s formula (Laplace, 1812), defined a century before Kolmogorov’s axioms were published (Kolmogorov, 1933). This classic definition states that the probability to observe a property *X* while raffling an element from a sample space *U* is defined by *P*(*X*)=|*X*|/|*U*|; (|*X*| being the number of elements fulfilling *X*, and |*U*| the size of the sample space). This means that a probability is the ratio of the number of “favourable cases” and of “all cases”. This, in turn, implies uniformity of the sample space, as we shall illustrate with an example.

If we choose a card from a shuffled 52-card set so that Laplace’s formula applies, we may work out the probability of any characteristic potentially associated with our card by dividing the number of cards that possess this particular characteristic by 52. For instance, the probability that the card is red is 0.5, because 26 cards are red and 26/52=0.5. But then we must have, for any particular card (e.g., an ace of diamond), a probability (*P*[ace of diamond]) of 1/52 given that there is only one such card. Any particular card thus has the same probability 1/52 to be chosen, a definition of the uniform probability on *U*.

A random variable has, thus, historically been defined as necessarily arising from uniformity, and uniformity is still an implicit assumption in the classroom. Although not all random variables in classical probability theory are uniform, there are always uniform variables at their root. To build non-uniform variables in a classical framework, one usually combines uniform variables. For instance, the sum of two dice, although not uniform, is a combination of two uniform variables. The maximum of two dice (i.e., the larger outcome) is another example. Now if you combine a random variable *X* with itself using subtraction, you get a deterministic variable, always equal to 0. This illustrates the fact that combining random processes may lead to a non-random process.

Additionally, since the work of Kolmogorov (1933), a more general view of random variables arose, which does not imply the requirement of uniformity as an essential property of every random phenomenon. This radical shift in the theory has led to an even stronger discrepancy between the notions of “random variable” and randomness itself. If any variable, even a constant, is called a “random variable”, how can we still relate such a “random” variable to randomness?

### Entropy

To overcome the confusion arising from this discrepancy, formal definitions of randomness have been suggested outside basic probability theory. A first attempt came from Shannon’s entropy:

Let *U* be a finite sample space (e.g., {0,1}), and *s* a sequence of elements of *U* (e.g., 000101101). The entropy of *s* is defined by *H*(*s*) = -Σ*P*(*x*) log_2_ (*P*(*x*)), where *P*(*x*) stands for the relative frequency of *x* (an element of *U*) in *s*. Entropy has in some cases been used as a measure of randomness (Giasu & Shenitzer, 1985), for instance, as a means to assess the quality of human pseudo-random production. We expect true randomness to exhibit maximal entropy.

Shannon’s entropy (Shannon & Weaver, 1949) is actually a measure of resemblance to uniformity (Giasu & Shenitzer, 1985), a feature that bears two consequences. First, entropy is maximal when the observed probability *P* on *U* (defined by *P*[*x*] being the relative frequency of *x*) is uniform. According to the maximal entropy definition of randomness, a truly random sequence must therefore conform to uniformity.

**Figure 1.**
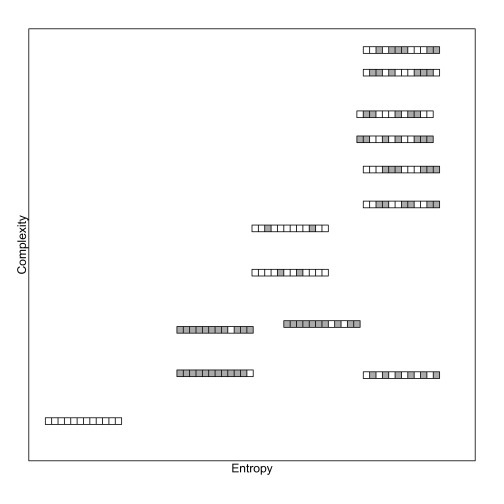
Examples of 12-item binary strings arranged according to their entropy and complexity. Each string is represented by an array of squares (grey squares for 1s and white squares for 0s). Although entropy and complexity are linked, some strings exhibit high entropy and low complexity, such as 010101010101 (bottom right).

Second, entropy only captures a specific property of a sequence s, namely the overall frequency of each outcome, irrespective of the sequence’s structure. For instance, the entropy of “0000011111” is exactly the same as that of “0110100101” or “0101010101”—and is maximal too—because the three strings have exactly the same number of 0s and 1s. This, of course, is not satisfactory if we seek a definition of randomness. The sequence “0101010101” should not be considered (very) random, because it is built according to a simple rule, and shows an excess of alternations too. Figure 1 displays some examples of binary strings, arranged in a two-dimensional space according to their entropy and algorithmic complexity (see next section).

Some authors suggest using *k*th-order (see next paragraph for a definition) entropy measures to better capture the structure of a sequence. However, as Grimsley, Monaghan and Wenstrup (2011) note, these measures are usually coupled with first-order entropy, and not used alone. But even if they were used alone, defining randomness by the maximization of a *k*th-order entropy would still imply that randomness is necessarily uniform:

To compute the entropy of a sequence like “001011” one considers the different symbols appearing in the sequence (i.e., 0, 0, 1, 0, 1, 1). Because the order of the symbols is not taken into account, entropy only depends on the frequency of 0s and 1s, irrespective of the structure. A solution to this problem is to turn our attention to bigrams (series of 2 consecutive symbols) instead of symbols. There are four possible bigrams, namely (00), (01), (10) and (11). In the previous case, “001011” would then be considered as a sequence of 3 bigrams: (00)(10)(11)^4^. The resulting entropy, called the second-order entropy of the sequence, does capture the local structure of the sequence. However, any sequence that maximizes second-order entropy will be balanced, so that the bigrams (00), (01), (10) and (11) appear with the same frequency. This in turn implies that the initial sequence is balanced in terms of 0s and 1s. In the same fashion, one may define third-order entropy using trigrams, or more generally *k*th-order entropy with *k*-grams. In each case, *k*th-order entropy captures some local structure of the sequence under consideration, but only some balanced sequences will maximize entropy (for a more formal account of *k*th-order entropy, see Cover & Thomas, 2006, p. 645, or Gray, 2011, p. 64).

Thus, a definition of randomness based on the idea that randomness will maximize either Shannon’s entropy or any *k*th-order entropy leads to the same result: Only balanced sequences (or uniform variables) can be considered random (although not all balanced sequences are random).

### Algorithmic complexity

Defining a random sequence as one that maximizes entropy leads to uniform sequences only—that is, it corroborates the intuition of equiprobability. However, the practice of using entropy as a measure of randomness has been criticized, and alternative measures have been suggested. The current theory of randomness is focused on the link between randomness and complexity. Algorithmic complexity (Li & Vitányi, 2008) is now the accepted foundation for a definition of a random series of symbols. It is nowadays widely used in biostatistics as a means to assess genetic complexity, and has interested psychologists lately as a possible explanation of pseudo-random human behaviour (see e.g., Gauvrit, Soler-Toscano, Zenil, & Delahaye, 2014; Tenenbaum, Kemp, Griffiths, & Goodman, 2011).

The algorithmic complexity of a finite sequence of digits is the length of the shortest program or algorithm that produces it and then halts. This length is defined with reference to an abstract computer: a universal (prefix-free) Turing Machine (UTM).

An infinite sequence *s*_1_*s*_2_…*s*_n_… is said to be random if and only if the complexity of the finite sequences *s*_1_*s*_2_…*s*_n_ remains large when *n* goes to infinity. More precisely, the complexity *K*(*s*_1_*s*_2_…*s*_n_) must satisfy the following property:

There exists a constant *c* > 0 such that *K*(*s*_1_*s*_2_…*s*_n_) *n* - *c* for all integers *n*.

The constant *c* depends on both the UTM and the sequence (*s*_i_), but not on *n*. One can understand the need for such a constant when we consider the fact that any random sequence bears local regularities. For instance, an infinite sequence of 0s and 1s contains long series of 0s. Such regularities can be exploited by the UTM, leading to a program which is shorter than “Print(*s*_1_…*s*_n_)”. A detailed account of this definition and its rationale is given in Li and Vitányi (2008).

This definition conveys two consequences. First, if a sequence is random, then the best algorithm that produces its first *n* terms (for any large enough *n*) is almost as long as the program “Print(*s*_1_*s*_2_…*s*_n_)” (the length of which is about *n*). Second, it can be formally demonstrated that a random sequence is necessarily balanced (uniform), because compression algorithms exist which are capable of taking advantage of any discrepancy from uniformity (Huffman, 1952).

This is a definition of a random (infinite) sequence, but may also be used to define a random process: A random process is a method that produces symbols such that when the method is repeated infinitely many times, the output is almost certainly (i.e., with a probability of 1) a random sequence.

An interesting feature of algorithmic complexity is that it bridges different points of view about randomness: A random sequence is at the same time complex, unpredictable, and passes every computable statistical test of randomness (Chaitin, 2004; Schnorr, 1973). As mentioned above, an intriguing feature of randomness as defined by algorithmic complexity is that uniformity is a precondition of randomness—that is, a sequence of heads or tails cannot be “random” if unbalanced (but not all balanced sequences are random). Once again, randomness implies uniformity.

### The combination paradox

Algorithmic complexity gives a formal definition of a random sequence or process, now widely accepted and used. This definition is an all-or-nothing rule: A sequence is either perfectly random, or not random. For this reason, in the following we will sometimes call a process “perfectly random” (rather than “random”) to indicate that it complies with the formal definition of a random process.

This definition yields an intriguing consequence: The combination of perfectly random processes is not necessarily perfectly random itself, an apparent contradiction. Throwing a fair die is a random process as defined by algorithmic complexity. When two dice are thrown and their results combined using a rule such as the sum, difference, product, or power, this results in non-uniform variables—that is, not perfectly random processes. To give another example, the maximum of two dice is not uniform. Indeed, the only situation giving a maximum of 1 is (1-1), whereas there are two situations (1-2 and 2-1) giving a 2, etc.

The product is not uniform either, since 1 can only be obtained from a 1-1 configuration, while 12 corresponds to 6-2, 2-6, 3-4, and 4-3. In the same vein, the two-dice problem illustrates the fact that a combination (sum) of uniform variables is not always uniform—or, in other words, that a combination of perfectly random processes needs not be perfectly random.

The well-known *law of large numbers* states that asymptotically, the observed frequencies equal their corresponding probabilities. For instance, throwing a die again and again will eventually result in having each number, say 6, exactly one sixth of the time. We may interpret this law as an even more striking example of the combination paradox: Combining infinitely many perfectly random and independent processes may result in a deterministic process.

## The psychological basis of the EB

In section 3 we described some tasks which have been found to elicit an incorrect “different outcomes are equally likely” response, and we have also given an overview of mathematical notions of randomness, and some controversies regarding mathematical approaches to this concept. Nevertheless, we have not discussed the following issues. First of all, how often do participants rely on the EB, and are these responses equally common in the case of different tasks? Do these responses have the same psychological/cognitive underpinnings?

With regard to the question of susceptibility to the EB, some of the tasks that we described above are notoriously difficult (for example, for the Monty Hall problem correct response rates of 3-21% have been reported, e.g., Burns & Wieth, 2004; De Neys & Verschueren, 2006; Friedman, 1998; Granberg & Brown, 1995; Krauss & Wang, 2003; Tubau & Alonso, 2003). Similarly, for the two children problem, Fox and Levav (2004) reported a correct response rate of 3.3% amongst MBA students with extensive training in statistics. Other tasks, such as the raffle problem turned out to be much less difficult. For example, Chiesi, Primi, and Morsanyi (2011) found correct response rates of 49-69% in developmental samples.

Making comparisons between the difficulty of various equiprobability tasks is not easy, as studies often focus on (different versions of) a single task (e.g., De Neys & Verschueren, 2006; Krauss & Wang, 2003), or different problems are administered to different groups of participants (e.g., Falk & Lann, 2008). Nevertheless, in a study where various tasks were included to measure the EB (Morsanyi et al., 2013), performance in the same group of participants varied widely across different tasks both in the case of “naďve” participants, and participants who completed a probability training (in Table 1 we present the rate of equiprobability and correct responses from the Morsanyi et al. (2013)study for some tasks that we described earlier). Given these differences in difficulty, we can expect that people who give an “equally likely” response to a certain task will not necessarily give this response to other tasks. Indeed, it seems that the “equally likely” response acts as a default (see Johnson-Laird et al., 1999) that educated adults tend to fall back on, if they encounter a task that involves probabilistic outcomes where a random generating process was involved, and they misrepresent the number and probability of potential outcomes (e.g., Falk & Lann, 2008; Fox & Levav, 2004). In the following sections we will describe two approaches which have been successfully applied to eliminate the EB in the case of various tasks.

### Eliminating the EB by changing the presentation format of tasks

One approach which has been successfully applied to eliminate the EB has been to create different versions of the problems, and find alterations which can make the problems easier to solve. This approach has been applied in the case of the Monty Hall problem (e.g., Fox & Levav, 2004; Krauss & Wang, 2003), the three cards problem (e.g., Falk & Lann, 2008), and the two children problem (e.g., Fox & Levav, 2004). These investigations (together with others, e.g., Falk, 1992; Johnson-Laird et al., 1999; Shimojo & Ichikawa, 1989) have revealed the following general steps of reasoning about probabilistic outcomes. Participants first identify the relevant cases given in the problem, and assign equal probabilities to these cases, unless otherwise stated in the description. Based on additional information, participants might add or eliminate cases. Finally, they assign equal probabilities to these updated cases (that is, the numerical value of equal probabilities is also updated, so that it corresponds to the updated number of cases).

This approach has also led to some suggestions regarding presentation formats and alterations to task content, which could help participants in avoiding the EB. Given the above analysis, these changes in presentation format were predominantly aimed at making it easier for participants to identify relevant cases (for example, in the case of the three cards problem), and to be able to correctly update the probabilities allocated to these cases when additional information is provided (e.g., in the Monty Hall problem) or to discard irrelevant information (e.g., in the three prisoners problem). Such manipulations include presenting the tasks in a frequency format (e.g., Krauss & Wang, 2003), using tree diagrams to compare probabilities (e.g., Johnson-Laird et al., 1999; Krauss & Wang, 2003), making individual cases more distinct by using names of human characters instead of using abstract labels (Falk & Lann, 2008), or by asking for a ranking instead of asking a question about the most likely outcome (e.g., Fox & Levav, 2004).

### Eliminating the EB through improving participants’ understanding of random processes

The other approach to address people’s difficulties has been to provide them with training. Fong and colleagues (Fong et al., 1986; Fong & Nisbett, 1991) successfully trained participants in the law of large numbers (which is required to solve the hospital problem, for example), both by providing examples, and by explaining participants about the law of large numbers. Fong and Nisbett (1991) found a training effect after a 2-week delay, and the training also generalized to problems that were related to a different domain (ability testing) as compared to the original training problems (sports content). These researchers also reported good memory for the law of large numbers (78.5% of their participants were able to recall this), which they contrasted with poor recall of the content of the problems that were used for training. Indeed, it seems that people find the idea that larger samples represent the parent population better than smaller ones intuitively compelling (Gravetter & Wallnau, 2009), and once the relevance of sample size is pointed out to them, they are able to apply the law of large numbers to solve probability problems.

The idea that participants have an implicit grasp of the law of large numbers has also been supported by a recent study. Building on a training procedure developed by Anway and Bennett (2004), Morsanyi et al. (2013) trained participants in the law of large numbers through exercises where random generating processes (such as throwing dice and flipping coins) were used to demonstrate the differences between the properties of large and small random samples. The exercises also included examples of outcomes with non-equal probabilities (see Morsanyi et al., 2013 for a more detailed description of the training procedure, and Table 1 for some of the results). Interestingly, whereas all participants improved on variants of the hospital problem (where they needed to rely on the law of large numbers), only participants with higher fluid intelligence improved on the other EB problems (including variants of the raffle problem and the two children problem). That is, participants with both high and low levels of fluid intelligence were able to grasp and apply the law of large numbers.

Another notable finding of this study is related to the idea of the law of small numbers. Tversky and Kahneman (1974) speculated that misconceptions regarding short sequences of random events arise because people generalize the properties of large samples to small samples. This is supposed to be based on the belief that errors in a random sequence cancel out each other. As we described above, Morsanyi et al. (2013) used training procedures where the properties of short and long sequences of probabilistic events were contrasted. Besides using variants of the hospital problem, they also used other test problems which assessed people’s ability to avoid misconceptions regarding short sequences. Interestingly, whereas the probability training improved participants’ performance on the hospital problem, these participants performed significantly worse on the other problems than controls. That is, although they were able to apply the law of large numbers when the task involved a direct comparison between short and long sequences of random events, their belief in the law of small numbers also increased. This clearly shows that being able to solve the law of large numbers problems does not require people to be able to correctly reason about the properties of short random sequences. At a more general level, besides their problems arising from the combination paradox, this example shows that people generally struggle with understanding when probabilistic outcomes should display “random properties”, such as the equiprobability of different outcomes or complexity.

### Two sources of the EB: Incorrect representations of potential outcomes and the combination paradox

Before we describe our account of the two main sources of people’s difficulties with some well-known EB tasks, we will briefly discuss the origin of the default assumption of equiprobability in the case of tasks including randomness. Most theorists assume that the equiprobability assumption stems from education in probability, as well as from more general concepts of equality and fairness (e.g., Falk & Lann, 2008). In our earlier discussion, we pointed out that simulations of probabilistic outcomes in the classroom typically involve equiprobable outcomes. As a result of being exposed to such examples, people develop the notion that random events are equiprobable “by nature” (Lecoutre, 1992). Indeed, both the theory about “naďve probability” (Johnson-Laird et al., 1999), and the “partition-edit-count” model (Fox & Levav, 2004) assume that once people identify probable outcomes, they will assign equal probabilities to these (unless they have good reasons to do otherwise). The claim that the equiprobability assumption arises from educational experiences is also supported by findings that the EB increases with age and probability education (e.g., Morsanyi et al., 2009).

In the previous sections we presented two approaches to eliminating the EB. One was to change the presentation format of tasks, so that people can more easily grasp the information regarding the number of cases and the individual probabilities of cases. This approach can help by eliminating the complexity and ambiguity inherent in some of the most difficult EB problems, such as the Monty Hall problem (e.g., Fox & Levav, 2004; Krauss & Wang, 2003), the three cards problem (Falk & Lann, 2008), and the two children problem (Fox & Levav, 2004). This approach typically works in the case of problems where there is a small number of cases/potential outcomes.

Although this approach has uncovered the sources of people’s difficulties with certain tasks, and could be useful in developing presentation formats which make it easier for people to reason about and understand probabilities, they cannot be used for improving people’s general understanding of probabilities.

The other approach has been to provide training in probabilities and, in particular, in the law of large numbers. This approach has been successful in the case of problems which include direct comparisons between short and long sequences of random events (Fong et al., 1986; Fong & Nisbett, 1991). Although these results are very important from an educational point of view, they have a relatively narrow scope. In the next section we will argue that education in the law of large numbers should be just one aspect of educating students about the “combination paradox”—that is, the fact that when random processes are combined, the outcome might be “less random” (i.e. more predictable; see also section 4.5).

### The combination paradox: Implications for probability education

In our discussion of mathematical approaches to randomness, we pointed out a particular property of random processes, which so far has not been addressed either by mathematicians or researchers of psychology. We have referred to this as the combination paradox. In our account, this phenomenon is a very important source of errors in probabilistic reasoning, and especially of the EB. Researchers have repeatedly claimed that the EB stems from a misunderstanding of randomness (e.g., Lecoutre, 1992; Morsanyi et al., 2009), but they have failed to elaborate on the nature of this misunderstanding. Throughout this paper, we have argued that people actually have a good intuitive grasp of the concept of randomness, which indeed involves uniformity. Nevertheless, they fail to appreciate the implications of the combination paradox.

Although one of the consequences of the combination paradox, the law of large numbers, is well-known, other consequences have not been clearly formulated. As a general rule, the unpredictability of outcomes decreases when we combine random processes, or, in general, when random processes go through transformations. As an example, consider a probabilistic sequence obtained by repeatedly throwing a drawing pin on a table. The drawing pin may land with pin up or down, but the probability of these outcomes is unequal. Basically, this is a biased heads-or-tails game. However, even if the result is skewed, an implicit assumption is that the drawing pin is thrown in a “fair way”. If a photograph of the pin was taken a few centimeters above the table while it falls, the assumption is that any position of the drawing pin would be just as likely as any other. Nevertheless, eventually we obtain a non-uniform probabilistic outcome from a (continuous) uniform underlying random variable. At the same time, according to the mathematical definition of a random binary series, the resulting infinite sequence of ups and downs is not perfectly random. Once again, the transformation reduces randomness. Discussing the implications of the combination paradox as part of probability education could be an important step forward.

A further important issue is that we know very little about people’s beliefs about the consequences of combining random processes, and the operations which can be performed on random processes. With regard to why people are misled by the combination paradox, a potential reason could be the application of the “same A same B” rule (Osman & Stavy, 2006) which is related to the acquisition and stabilization of the proportionality schema (see, e.g., Fischbein & Schnarch, 1997; Van Dooren, De Bock, Hessels, Janssens, & Verschaffel, 2005). For example, Mendel (1998) presented students with a problem in which two rectangles were shown. Students were told that the second rectangle is a modified version of the first one where the length of the rectangle was decreased by 20% and the width was increased by 20%. Students were asked about the perimeters of the two rectangles (i.e., whether they were equal, or whether the perimeter was longer in the case of one of the rectangles). In this experiment participants had knowledge about the relevant rule (i.e., how to compute the perimeter of a rectangle), and the available perceptual information was also in line with the rule (i.e., that the perimeter of rectangle 1 was longer). Nevertheless, over 70% of the students applied the “same A - same B” rule. That is, they claimed that the perimeters of the two rectangles were equal, because “adding 20% and removing 20% equals to no change.” Similarly, when combining random processes, students might have the impression that given that the generating processes are fair and unpredictable, the outcome should be fair and unpredictable, too.

Investigations into people’s beliefs about the consequences of combining random processes could be a fruitful avenue for furthering our understanding of how and why people might make mistakes when they reason about probabilities. This research should also form the basis of novel educational and training approaches.

## Concluding comments

The EB leads to systematic errors in probability judgments. It is a consequence of an intuition which develops as a result of probability education (Morsanyi et al., 2009). It has long been considered as a conceptual error about randomness—that participants wrongly attribute uniformity to perfectly random processes. Nevertheless, this claim is not coherent with current mathematical theory: Randomness does indeed imply uniformity. Thus, when participants commit the EB, their problem is not that their understanding of the concept of randomness is fundamentally mistaken.

In the present paper we have provided an overview of both mathematical and psychological approaches to the EB. As a result of our analysis, we have identified a gap, which affects both literatures. This analysis suggests that one particular property of randomness might contribute greatly to the pervasiveness of the EB. Indeed, as opposed to what we would naturally think, the combination of random processes is not necessarily uniform (and therefore not necessarily perfectly random), or, more generally, certain transformations change the essential properties of random sequences. Thus, when people erroneously apply the EB, they *correctly* assume that randomness includes equiprobability. What they do not recognize is that the probabilistic outcomes that they are faced with are *not perfectly random*.

Whereas training in the law of large numbers might be effective in the case of certain EB tasks, and it has been used both by researchers and educators, to the best of our knowledge, no research has addressed so far people’s beliefs about the outcome of combining random processes. Thus, it is important that future studies assess these misconceptions and test different training approaches which could help in tackling them.

In summary, this paper was aimed at both highlighting the pervasiveness of the EB in the domain of probabilistic reasoning, and identifying the cognitive processes underlying it. We have argued that the EB typically emerges in two situations. One is when people incorrectly assign equal probabilities to individual cases. The other one is when people erroneously assume that combining random processes will leave the “random properties” of those processes unaffected. The bulk of research so far has focused on cases where people experience difficulties with regard to correctly assigning probabilities to individual cases (e.g., Johnson-Laird et al., 1999; Falk & Lann, 2008; Fox & Levav, 2004; Krauss & Wang, 2003). Although this research is important with regard to developing more effective ways of presenting probabilistic information, it has little to say about how people generally reason about probabilistic outcomes. The purpose of the present paper was to draw attention to another main source of difficulty regarding probabilistic reasoning. Our hope is that our discussion will inspire new research in this area, and will inform the development of novel training procedures.

## Footnotes

1 Calling the EB a bias is certainly arguable and one may wish to use a more neutral expression such as “equiprobability answer”. In the following, we will consider EB and “equiprobability answer” as equivalent.

2 These psychological reasons may rely on heuristics too, and may be incorrect from a mathematical perspective (see e.g., Teigen & Keren, 2007).

3 The equivalence of the problem of three prisoners and the Monthy Hall dilemma becomes clear when one identifies each door (in the Monthy Hall dilemma) with a prisoner (in the problem of three prisoners), and the reward with the fact that the prisoner will be spared. The probability of winning for prisoner C (or by picking door C) is unchanged when the new information that B will not be set free (or that door B hides no reward) is given, but at the same time the probability that prisoner A will be spared (or that door A hides the reward) increases to 2/3.

4 Another version uses overlapping bigrams, and would associate the following bigrams with the sequence “001011”: (00)(01)(10)(01)(11). This does not alter the results discussed in the following.

5 Morsanyi et al. (2013) included 6 problems in their study, which were designed to measure the EB (performance on individual tasks was not reported).
